# Editorial: Prospecting microbial technologies for agricultural sustainability

**DOI:** 10.3389/fbioe.2026.1885899

**Published:** 2026-07-16

**Authors:** Damini Maithani, Divjot Kour, Sandeep K. Malyan, Abhay Kumar

**Affiliations:** 1 Natural Resource Management: Plant Microbiology, College of Horticulture, Sardar Vallabhbhai Patel University of Agriculture and Technology, Meerut, India; 2 University Centre for Research and Development, Chandigarh University, Gharuan, Punjab, India; 3 Department of Environmental Studies, Dyal Singh Evening College, University of Delhi, New Delhi, India; 4 Institute of Environmental Engineering, Polish Academy of Sciences, Zabrze, Poland; 5 CaptureBank AS, Grimstad, Norway

**Keywords:** biofertilizers, disease diagnostics, nano-biosensors, plant disease management, sustainable agriculture

The growing global population, rapid climate change, shrinking arable lands, and environmental degradation collectively threaten agricultural productivity and food security worldwide (Kumar et al. 2024). Conventional agricultural practices relying heavily on synthetic fertilizers, pesticides, and intensive land management have significantly increased crop productivity over recent decades. However, excessive dependence on these inputs has also contributed to soil degradation, environmental pollution, and adverse effects on human health (Saha et al.2024). In this regard microorganisms with plant growth promoting and stress mitigating traits offer a promising non-toxic and eco-friendly solution for transforming modern agriculture (Romão et al. 2025).

Microorganisms associated with soil and plants play fundamental roles in nutrient cycling, soil fertility maintenance, phytohormones production, stress mitigation, disease suppression, degradation of organic matter, and improving ecosystem stability (Nabi, 2023). Potential microbial formulations can be utilized as biofertilizers and biopesticides as a suitable alternative to agrochemicals for promoting plant growth and combating persisting Research Topic of climate change and food security (Soares, 2022; Shahzad et al. 2025). Advances in microbial ecology, biotechnology, and bioengineering have enhanced our understanding of plant-microbe interactions and opened new opportunities for developing sustainable agricultural practices.

Although microorganisms-based solutions are gaining attention by scientific community, there are still some challenges limiting their scalability which need to be understood and resolved by standardizing formulations using modern microbial technologies, using consortia instead of single strains, addressing knowledge gaps and designing suitable policy frameworks (Shahzad et al.2025). This Research Topic focuses on innovative studies addressing the evaluation, development, and application of microbial technologies for soil health restoration and environmentally sustainable agriculture. The contributions published in this issue collectively highlights the expanding role of beneficial microorganisms in improving soil fertility, enhancing crop productivity, mitigating environmental stress, and suppressing use of agro-chemicals. The Research Topic includes six original research articles and two review articles on different Research Topic, including microbial technologies and their impact on rhizosphere engineering, soil fertility, plant growth promoting bacteria (PGPB), microbial diversity, nano-biosensors technologies and their use in plant disease diagnostics, microbial interventions in fermentation, and waste valorization. Studies also demonstrated role of microbial consortia and engineered microbial communities in enhancing agricultural productivity and ecosystem sustainability.

The original research by Li et al. focused on the isolation, screening, and identification of microorganisms exhibiting multiple plant growth promoting traits, including phosphate solubilization, indole-3- acetic acid production, nitrogen fixation, siderophore production, and evaluated their impact on soil health restoration and yield enhancement of rice cultivated in non-grain converted land. The authors identified promising bacterial strains as *Enterobacter hormaechei* and *Yokenella regensburgei*, enhancing soil fertility and rice growth in degraded agricultural land. The efficiencies of these strains were further assessed through pot trials, Correspondingly, improvements were observed growth parameters and available soil phosphorous levels. High throughput sequencing revealed substantial reshaping of the soil microbial community, driven by alteration in soil organic matter, pH, iron and phosphorus availability. The study demonstrated the potential application of these bacterial strains in restoring marginal and degraded agricultural lands, thereby facilitating sustainable rice cultivation and broader grain crops production.

Shikha et al. comprehensively reviewed the evolution of plant disease diagnostics from conventional pathogen detection methods to advanced biosensing technologies. The study highlighted the significance of biosensor-based platforms for rapid, precise, early-stage detection of plant diseases, contributing to sustainable and eco-friendly crop protection through minimized chemicals inputs. The review provides a detailed overview of various classes of biosensors, their integration with nanotechnology, and a comparative evaluation of traditional *versus* advanced disease diagnostic systems for sustainable crop production.

Another original research contribution by Yao et al. investigated the impact of *Staphylococcus saprophyticus* inoculation on the fermentation characteristics and aroma profile of cigar filler tobacco leaves. The Inoculation significantly improved the volatile compound profile and increased number of beneficial microorganisms, particularly *Bacillus* sp. Metabolic pathways analysis showed upregulation of enzymatic sugar conversion reactions confirming enhanced microbial metabolism during fermentation. The study concluded that the application of exogenous microorganisms could substantially contribute in improving both the fermentation process and quality attributes of tobacco product.

In a related study, Yao et al. reported the positive effect of tobacco flower bud extract on reshaping the microbial community during cigar fermentation. The treatment effectively suppressed pathogenic microorganisms such as *Aspergillus* sp., while promoting the proliferation of beneficial microbial population. This microbial shift was observed to facilitate carbohydrate and protein degradation along with a reduction in nicotine content. Aroma profiling analysis demonstrated an increase in desirable aromatic compounds, whereas metagenomic functional analysis revealed inhibition of toxic metabolite biosynthetic pathways. The study further highlighted the potential of tobacco flower bud extracts as sustainable bio-based additives for improving cigar fermentation and promoting agricultural waste valorization.

In a study by Lan et al., the effect of rain shelter structures on soil microbial dynamics, fruit quality, and yield performance in kiwifruit production systems was evaluated. Research findings demonstrated that rain shelter cultivation positively improved fruit taste profile, physical attributes, and nutritional quality compared with conventional open field cultivation. Moreover, rain shelter cultivation positively influenced microbial populations, enzymatic activities, and microbial biomass. The study concluded that soil microbial communities and associated enzymatic processes contribute in regulating kiwifruit productivity and nutritional quality under protected rain shelter cultivation.

Prajapati et al. synthesized gold nanoparticles (Au-NPs) using extracellular α-amylase produced by *Bacillus subtilis* VSP4 under solid state fermentation. The work demonstrated improved α-amylase production and efficient Au-NPs synthesis through the application of statistical optimization tools, specifically. These methods reduce time and operational cost of the fermentation process in comparison to traditional methodologies, underscoring the industrial and biotechnological potential of microorganism mediated nanoparticle synthesis.

Narware et al. reviewed recent advances in nanotechnology-driven real time monitoring and plant disease diagnostic systems. The review highlighted the structural and functional components of nano-biosensors and nano-enabled detection platforms for rapid pathogen identification. Comparative overview between conventional diagnostic methods and nano-biosensor technologies revealed substantial advantages of this approach in terms of speed, sensitivity, portability, and detection efficiency. Furthermore, the study critically discussed existing challenges associated with nano-enabled technologies, including toxicity concerns, environmental compatibility, long-term durability, and operational performance.

Sharma et al. investigated the biofertilizer and biocontrol potential of *Stentrophomonas maltophila* in wheat cultivation systems. Seed priming with *S. maltophila* significantly enhanced germination rate, plant growth, and yield-related parameters under both biotic and abiotic stress conditions. The persistence and viability of the introduced strain within the rhizosphere were further validated through metagenomic approach. The study discussed and concluded that *S. maltophila* possesses considerable potential as an eco-friendly biofertilizer and biocontrol agent for sustainable wheat production under stress-prone agricultural environments.

Collectively, these studies demonstrate a growing convergence between microbial biotechnology, nanotechnology, and precision agriculture, highlighting an important shift toward integrated and climate-resilient farming systems.

This Research Topic will stimulate further research and foster collaborative efforts toward harnessing microbial resources for sustainable agriculture and environmental stewardship. Future agricultural sustainability will increasingly depend on our ability to understand, manage, and engineer beneficial microbial communities. Continued research in microbial technologies, microbiome engineering, and eco-friendly agricultural innovations will be instrumental in building climate-resilient and resource-efficient agricultural systems capable of meeting future global food demands while preserving environmental integrity. Future progress will depend not only on technological innovation but also on effective field validation, optimized bioformulations, farmer accessibility, regulatory support, effective policy framework and its implementation into existing agricultural systems.

## References

[B1] Kumar A. Saharan B. S. Parshad J. Gera R. Choudhary J. Yadav R. (2024). Revolutionizing Indian agriculture: the imperative of advanced biofertilizer technologies for sustainability. Discov. Agric., 2(1), 24.

[B2] Nabi M. (2023). Role of microorganisms in plant nutrition and soil health. In Sustainable Plant Nutrition (pp. 263–282). Academic Press.

[B3] Romão I. R. do Carmo Gomes J. Silva D. Vilchez J. I. (2025). The seed microbiota from an application perspective: an underexplored frontier in plant–microbe interactions. Crop Health, 3(1), 12. 41649661 10.1007/s44297-025-00051-6 PMC12825913

[B4] Saha B. Fatima A. Saha S. Sahoo S. K. Poddar P. (2024). Environmental pollution due to improper use of chemical fertilizers and their remediation. In Environmental Contaminants (pp. 203–219). Apple Academic Press.

[B5] Shahzad M. Hayat R. Mujtaba G. Rehman W. U. Nadeem M. (2025). Biofertilizers in sustainable agriculture: mechanisms, applications, and future prospects. Discov. Agric., 3(1), 224.

[B6] Soares E. V. (2022). Perspective on the biotechnological production of bacterial siderophores and their use. Appl. Microbiol. Biotechnol., 106(11), 3985–4004. 35672469 10.1007/s00253-022-11995-y

